# Prenatal human skin expresses the antimicrobial peptide RNase 7

**DOI:** 10.1007/s00403-013-1340-y

**Published:** 2013-04-02

**Authors:** Christopher Schuster, Regine Gläser, Christian Fiala, Wolfgang Eppel, Jürgen Harder, Jens-M. Schröder, Adelheid Elbe-Bürger

**Affiliations:** 1 Division of Immunology, Allergy and Infectious Diseases (DIAID), Department of Dermatology, Laboratory of Cellular and Molecular Immunobiology of the Skin, Medical University of Vienna, Währinger Gürtel 18-20, 1090 Vienna, Austria; 2 Department of Dermatology, University Hospital Schleswig-Holstein, Campus Kiel, Kiel, Germany; 3 Gynmed Ambulatorium, Vienna, Austria; 4 Department of Gynaecology and Obstetrics, Medical University of Vienna, Vienna, Austria

**Keywords:** Antimicrobial peptides, Prenatal, Skin, Ontogeny

## Abstract

Antimicrobial peptides and proteins (AMPs) play important roles in skin immune defense due to their capacity to inhibit growth of microbes. During intrauterine life, the skin immune system has to acquire the prerequisites to protect the newborn from infection in the hostile environment after birth, which includes the production of skin AMPs. The aim of this study was to analyze the expression of RNase 7, HBD-2/3 and psoriasin during human skin development, thus, providing a deeper insight about the maturity of a fundamental component of the innate immune system. We found low RNase 7 expression levels in the periderm but no expression of HBD-2/3 and psoriasin in first trimester human skin using immunohistochemistry. At the end of the second trimester, RNase 7 is expressed weakly in all epidermal layers with a marked signal in the stratum corneum. HBD-3 and psoriasin are focally expressed while HBD-2 is not detectable. Analysis of supernatants from cultured prenatal skin cells showed that in contrast to adult control, RNase 7 and psoriasin are not found in prenatal skin, suggesting that AMPs are detectable but are not secreted. This study shows the differential expression of AMPs in developing, non-perturbed human prenatal skin. It is conceivable that the combined expression of RNase 7, HBD-3 and psoriasin in fetal skin constitutes a developmental program to exert a broad spectrum of antimicrobial activity to maintain sterility in the amniotic cavity.

## Introduction

Antimicrobial peptides and proteins (AMPs) play important roles in skin immune defense due to their capacity to inhibit growth of microbes by either direct killing or modification of local immune responses [18]. Healthy adult human skin contains a variety of pharmacological as well as functionally divergent AMPs that interact in preventing skin infections, including RNase 7, human β-defensin (HBD)-1 and psoriasin [21]. Conversely, HBD-2 and 3 and cathelicidins (LL-37) are locally induced in keratinocytes upon inflammation and infection, but are otherwise not or barely detectable [10, 17]. To date, the acquisition of AMPs in prenatal human skin is largely unknown [12]. During prenatal development, the epidermis matures from a single layered epithelium covered by a developmental specific structure, the periderm [1], to the multilayered, keratinized epidermis at the end of the second trimester [6]. Given the broad antimicrobial activity of skin-derived AMPs, it can be hypothesized that during in utero development high levels of skin AMPs should be found to maintain the sterility of the amniotic cavity and to prevent microbial invasion [5]. The significantly higher levels of cathelicidins and HBD-2 in neonatal human foreskin compared to adult skin corroborate this notion [3]. In addition, the constitutive expression of HBD-1 in non-infected fetal skin at 21–24 estimated weeks of gestation (EGA) suggests the existence of at least one kind of antimicrobial protection during skin development. Intriguingly, chorioamnionitis following microbial invasion of the amniotic cavity may extend into fetal dermatitis [12], which, in turn, leads to an upregulation of HBD-2 and 3. Taken together, these data support the growing body of evidence that already in utero rapid innate immune responses occur in the skin that aim at maintaining sterility in the amniotic cavity.

AMPs have also been identified in the vernix caseosa, a lipid-rich substance covering the human fetus and neonate [15, 23]. The vernix is composed of sloughed off periderm cells and keratinocytes as well as sebaceous gland products and acts as a mechanical barrier between fetal skin and the amniotic cavity. High levels of human neutrophil defensins (HNP)1–3 and to a lower extent lysozyme, LL-37, ubiquitin and psoriasin were detected, suggesting that the vernix also has antimicrobial properties. Whether these proteins originate from sparse neutrophils in the amniotic cavity and/or the developing skin is unknown so far [5, 16].

The expression of RNase 7, HBD-2/3 and psoriasin in embryonic and fetal skin has not been investigated in detail [12]. Yet, expression of these AMPs in the developing epidermis and their potential role in preventing microbial invasion of the amniotic cavity remains unknown [5]. The aim of this study was to analyze the expression and temporal development of various AMPs during skin development to gain a deeper insight about the maturity of a fundamental component of the innate immune system.

## Materials and methods

After legal termination of pregnancy, six specimens of human embryonic and fetal trunk skin ranging from 10 to 24 weeks EGA were studied. The age was estimated by crown-rump length and maternal history. Healthy adult and psoriatic skin was collected as positive control. The study was approved by the local ethics committee and conducted in accordance with the declaration of Helsinki Principles. Parents/participants gave their written informed consent.

Embryonic, fetal and adult skin specimens were fixed in formalin and embedded in paraffin. Five micrometer sections were cut, deparaffinized, rehydrated, and blocked with normal rabbit serum. The sections were then incubated with polyclonal antisera against RNase 7 and HBD-2/3 or a monoclonal antibody against psoriasin. Specific binding was then detected using biotinylated IgG antibody to rabbit, mouse and goat, respectively (Vector Laboratories, Burlingame, CA), followed by incubation with vector red and counterstaining with hematoxylin.

After removal of subcutaneous tissue, fetal and adult skin was incubated on 1.2 U/ml Dispase II (Roche Diagnostics, Indianapolis, IN) in PBS overnight at 4 °C. It was not possible to efficiently separate dermis and epidermis in fetal skin, thus, unseparated skin regardless of age was vigorously agitated in a shaking bath in 0.53 U/ml Liberase 3 (Roche Diagnostics, Indianapolis, IN) in PBS at 37 °C for 60–90 min. The resulting single cell suspensions were cultured for 48 h (10^6^ cells/ml) in RPMI 1640 medium (Invitrogen, Eugene, OR) supplemented with 10 % heat-inactivated FCS (PromoCell, Heidelberg, Germany), 25 mM HEPES, 10 μg/ml gentamicin, 2 mM l-glutamine, 0.1 mM non-essential amino acids, 1 mM sodium pyruvate, 50 μM 2-mercapto-ethanol, and 0.002 % antibiotic-antimycotic solution (all Invitrogen). After 48 h, supernatants were harvested and stored at −80 °C until use. RNase 7, HBD-2/3 and psoriasin levels were analyzed by ELISA as described previously [9, 13, 22]. Experiments were performed in triplicates.

Differences between groups were assessed with the Mann–Whitney *U* test (GraphPad Software, San Diego, CA). The reported *p* value is a result of a two-sided test. A *p* value <5 % is considered statistically significant.

## Results

**Fig. 1:**
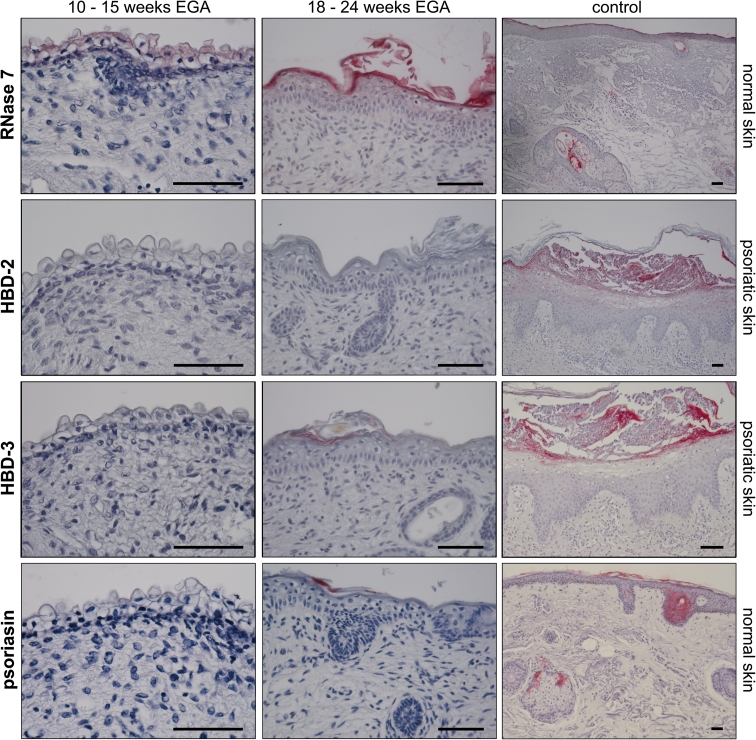
RNase 7 but not HBD-2/3 and psoriasin are expressed in prenatal human epidermis. Immunohistochemical staining was performed on paraffin sections of fetal and adult skin and RNase 7, HBD-2/3, and psoriasin binding was visualized using vector red. Normal adult skin or psoriatic lesional skin served as positive controls. *Scale bars* 50 μm

During intrauterine life, the skin immune system has to acquire the prerequisites to protect the newborn from infection in the hostile environment after birth, which include the production of skin AMPs. Using immunohistochemistry, we found that RNase 7 stains faintly the cytoplasm of periderm cells but not neighboring keratinocytes, developing skin appendages, or dermal cells between 10 and 15 weeks EGA. However, embryonic skin does not express HBD-2/3 and psoriasin (Fig. 1, left column; *n* = 3).

With advancing gestational age, the staining pattern changes. After the disintegration of the periderm, RNase 7 is expressed weakly in all epidermal layers with a marked signal in the stratum corneum and hair follicles, similar to healthy adult skin (Fig. 1, right column; *n* = 3). Focal expression of both HBD-3 and psoriasin in the developing stratum corneum is observed in one sample. HBD-2 is untraceable in all samples investigated (Fig. 1, middle column; *n* = 3).

**Fig. 2:**
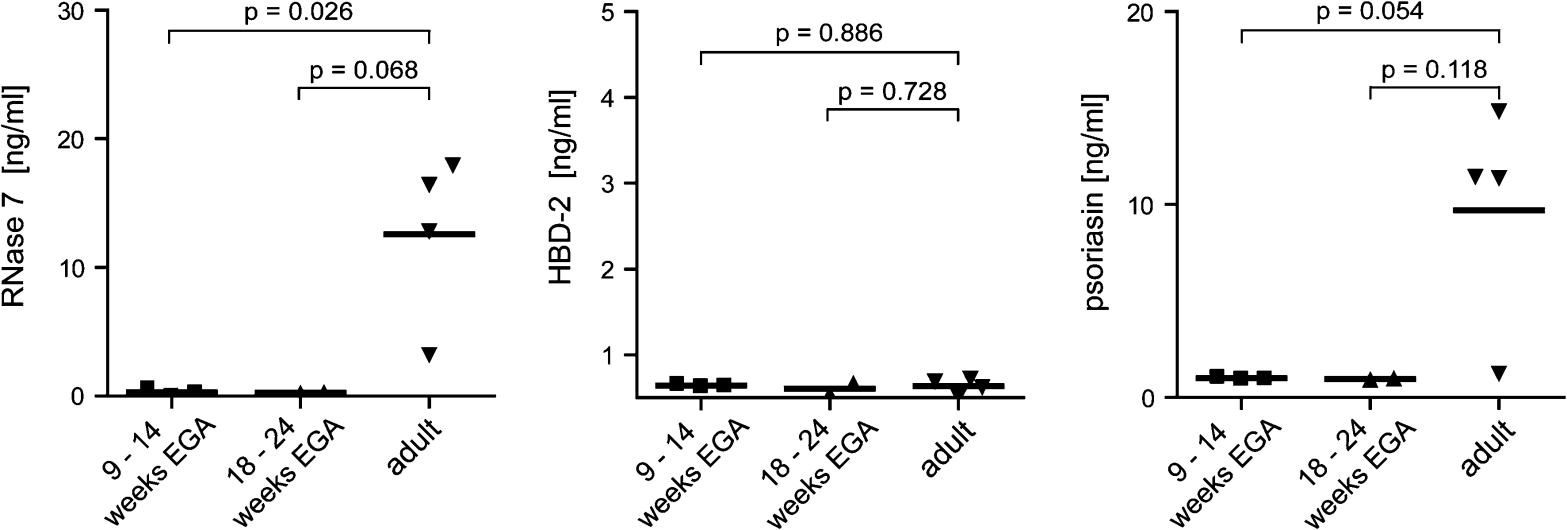
Supernatants of prenatal human skin secrete lower levels of AMPs than adult skin. Single cell suspensions of embryonic, fetal, and adult skin were cultured for 48 h, supernatants were harvested, and RNase 7, HBD-2 and psoriasin levels were analyzed by ELISA. *Bars* represent the mean

To further assess the capacity of fetal keratinocytes to produce and secrete AMPs and to confirm the immunohistochemical staining, supernatants of 48 h cultured single cell suspensions were analyzed for RNase 7, HBD-2/3 and psoriasin by ELISA (Fig. 2). Levels of HBD-2 are similar among the investigated groups, while HBD-3 is not detectable at all. Surprisingly, higher levels of both RNase 7 and psoriasin were found in supernatants of adult skin compared to prenatal skin, suggesting that fetal keratinocytes may be inferior to secrete AMPs than adult keratinocytes.

## Discussion

This study shows the differential expression of AMPs in developing, non-perturbed prenatal human skin, indicative of a controlled developmental process and possibly a prerequisite to maintain sterility of the amniotic cavity. At all investigated developmental age groups, staining was exclusively found in the epidermis or skin appendages, but not in the dermis. Though detectable in the epidermis, AMPs are not secreted into fetal cell culture supernatants.

AMPs in human skin can be classified according to their constitutive (e.g. RNase 7, psoriasin) or inducible expression (HBD-2/3) [2, 18]. After 18 weeks EGA, RNase 7 and—in one specimen—psoriasin were detectable in a pattern similar to adult skin. Conversely, HBD-2 was not detectable in developing skin from 10–24 weeks EGA, while HBD-3 was focally expressed in the stratum corneum in one donor at 22 weeks EGA, confirming previously reported results by Kim et al. [12]. It is conceivable that the combined expression of RNase 7, HBD-3 and psoriasin in fetal skin constitutes a developmental program to exert a broad spectrum of antimicrobial activity to maintain sterility in the amniotic cavity.

The sterility of the amniotic cavity is, under physiologic condition, guaranteed by the cooperation of AMPs in the amniotic fluid and a complex physical barrier composed of chorioamniotic membranes, cervical epithelium, and the cervical plug preventing microbe ascension [5]. With regard to AMPs, HNP 1–3 and bactericidal/permeability increasing protein can be identified in amniotic fluid starting from 14 weeks EGA, probably derived from leukocytes of fetal origin [5, 16]. Our finding that RNase 7, HBD-3 and psoriasin are not detectable in cell culture supernatants may suggest that these peptides are simply not secreted and, thus, may not be present in the amniotic fluid. In skin washing fluids of healthy adult human skin RNase 7 is readily detectable, suggesting that it is secreted and functions primarily outside the keratinocyte [21]. As fetal skin between 18 and 24 weeks EGA shows a similar staining pattern as adult skin but no significant secretion of RNase 7, we conclude that the secretion of RNase 7 may be immature. Yet, that does not preclude an antimicrobial effect, given that periderm cells and corneocytes containing these peptides are sloughed off to form the vernix caseosa, where they could be biologically active. Accordingly, it has recently been described that vernix caseosa from neonates born at term contains AMPs including psoriasin and HNP 1–3 [15, 23]. Based on our immunohistochemical data, we propose that psoriasin found in the vernix caseosa is of epidermal origin. Moreover, it can be assumed that corneocytes containing RNase 7, HBD-3 and psoriasin are trapped in the vernix and, in addition to the physical barrier, provide a further chemical barrier to prevent perinatal skin infection.

The conspicuous presence of RNase 7 in periderm cells was unexpected, underscoring the fact that periderm cells differ from neighbouring keratinocytes. It is, thus, conceivable, that RNase 7 plays apart from its ancestral function as an AMP a role in the differentiation and development of the primitive epidermis. Whether AMPs as ancient members of the innate immune system contribute in developmental processes is not known so far [24]. Knockout mice could be useful in the elucidation of potential functions of AMPs in the developing epidermis.

The presence of AMPs in the second trimester skin confirms the maturity of the epidermis at the end of the second trimester, given that not only all relevant structural and differentiation molecules are in place but also various AMPs and—though in lower numbers—Langerhans cells [1, 12, 14, 19]. This corroborates the notion that the innate arm of the skin immune system is in place when the limit of viability is reached at around 22–24 weeks EGA.

In summary, the present study identified for the first time expression of selected AMPs in developing human skin, corroborating both the structural maturity of the skin and the advanced state of the innate immune system at the end of the second trimester [4, 7, 8, 11, 19, 20].

## References

[1] Akiyama M, Smith LT, Yoneda K (1999). Periderm cells form cornified cell envelope in their regression process during human epidermal development. J Invest Dermatol.

[2] Braff MH, Bardan A, Nizet V (2005). Cutaneous defense mechanisms by antimicrobial peptides. J Invest Dermatol.

[3] Dorschner RA, Lin KH, Murakami M (2003). Neonatal skin in mice and humans expresses increased levels of antimicrobial peptides: innate immunity during development of the adaptive response. Pediatr Res.

[4] Elbe-Bürger A, Schuster C (2010). Development of the prenatal cutaneous antigen-presenting cell network. Immunol Cell Biol.

[5] Espinoza J, Chaiworapongsa T, Romero R (2003). Antimicrobial peptides in amniotic fluid: defensins, calprotectin and bacterial/permeability-increasing protein in patients with microbial invasion of the amniotic cavity, intra-amniotic inflammation, preterm labor and premature rupture of membranes. J Matern Fetal Neonatal Med.

[6] Foster CA, Bertram JF, Holbrook KA (1988). Morphometric and statistical analyses describing the in utero growth of human epidermis. Anat Rec.

[7] Foster CA, Holbrook KA (1989). Ontogeny of Langerhans cells in human embryonic and fetal skin: cell densities and phenotypic expression relative to epidermal growth. Am J Anat.

[8] Fujita M, Furukawa F, Horiguchi Y (1991). Regional development of Langerhans cells and formation of birbeck granules in human embryonic and fetal skin. J Invest Dermatol.

[9] Gläser R, Harder J, Lange H (2005). Antimicrobial psoriasin (S100A7) protects human skin from escherichia coli infection. Nat Immunol.

[10] Hata TR, Gallo RL (2008). Antimicrobial peptides, skin infections, and atopic dermatitis. Semin Cutan Med Surg.

[11] Iram N, Mildner M, Prior M (2012). Age related changes in expression and function of toll-like receptors in human skin. Development.

[12] Kim YM, Romero R, Chaiworapongsa T (2006). Dermatitis as a component of the fetal inflammatory response syndrome is associated with activation of toll-like receptors in epidermal keratinocytes. Histopathology.

[13] Köten B, Simanski M, Gläser R (2009). RNase 7 contributes to the cutaneous defense against *Enterococcus faecium*. PLoS ONE.

[14] Lee SC, Lee JB, Kook JP (1999). Expression of differentiation markers during fetal skin development in humans: immunohistochemical studies on the precursor proteins forming the cornified cell envelope. J Invest Dermatol.

[15] Marchini G, Lindow S, Brismar H (2002). The newborn infant is protected by an innate antimicrobial barrier: peptide antibiotics are present in the skin and vernix caseosa. Br J Dermatol.

[16] Sampson JE, Theve RP, Blatman RN (1997). Fetal origin of amniotic fluid polymorphonuclear leukocytes. Am J Obstet Gynecol.

[17] Schröder JM (2011). Antimicrobial peptides in healthy skin and atopic dermatitis. Allergol Int.

[18] Schröder JM, Harder J (2006). Antimicrobial skin peptides and proteins. Cell Mol Life Sci.

[19] Schuster C, Vaculik C, Fiala C (2009). HLA-DR^+^ leukocytes acquire CD1 antigens in embryonic and fetal human skin and contain functional antigen-presenting cells. J Exp Med.

[20] Schuster C, Vaculik C, Prior M (2012). Phenotypic characterization of leukocytes in prenatal human dermis. J Invest Dermatol.

[21] Simanski M, Köten B, Schröder JM (2012). Antimicrobial RNases in cutaneous defense. J Innate Immun.

[22] Voss E, Wehkamp J, Wehkamp K (2006). NOD2/CARD15 mediates induction of the antimicrobial peptide human beta-defensin-2. J Biol Chem.

[23] Yoshio H, Tollin M, Gudmundsson GH (2003). Antimicrobial polypeptides of human vernix caseosa and amniotic fluid: implications for newborn innate defense. Pediatr Res.

[24] Zasloff M (2002). Antimicrobial peptides of multicellular organisms. Nature.

